# Screening cells for crystals: a synergistic approach

**DOI:** 10.1107/S1600576720014971

**Published:** 2020-11-17

**Authors:** Terese Bergfors, Soneya Majumdar

**Affiliations:** a Department of Cell and Molecular Biology, Uppsala University, Sweden

**Keywords:** *in cellulo* crystallization, *in vivo* crystals, small-angle X-ray scattering, X-ray powder diffraction

## Abstract

Lahey-Rudolph and co-workers [*J. Appl. Cryst.* (2020), **53**, 1169–1180] have reported a rapid and sensitive method to screen for crystals *in cellulo* – a welcome addition to the structural biology toolbox.

*In cellulo* crystallization – the formation of crystals inside living cells – is an intriguing phenomenon. How does nature manage to produce *in vivo* crystals while crystallization in the laboratory is so difficult? An understanding of nature’s own crystallogenesis would expand our possibilities to use this process. However, one difficulty hampering the study of *in vivo* crystals is detecting them inside their host cells. Spatial constraints limit the crystal size and the chaotic background of the host cell gives a high signal-to-noise ratio. In the October 2020 issue of *Journal of Applied Crystallography*, Lahey-Rudolph *et al.* (2020 ▸) tackled these problems by combining small-angle X-ray scattering (SAXS) with X-ray powder diffraction (XRPD). Their work has resulted in a rapid and sensitive method to screen for crystals *in cellulo*.

*In cellulo* crystallization occurs in all kingdoms of life. Plants, viruses, insects, yeast, fungi and mammals use it as a way to store proteins, to protect them from proteolysis and to make solid-state catalysts (Schönherr *et al.*, 2018 ▸). *In vivo* crystals can also result from non-native conditions, for example during heterologous overexpression or some pathologies. XRPD was used early on to characterize the unit cells of *in vivo* crystals, for example from *Bacillus thuringiensis* (Li *et al.*, 1988 ▸). But it is thanks to increased availability of free-electron lasers and fourth-generation synchrotrons that *in vivo* and other nano- and microcrystals can now be used for diffraction studies with serial femtosecond X-ray crystallography or microfocus beams.

*In vivo* crystals can be recovered for diffraction by lysing the host cells, but this often affects the crystal quality (Gallat *et al.*, 2014 ▸). It would be better to diffract the crystals while still *in cellulo*, and in a proof-of-concept experiment, Axford *et al.* (2014 ▸) did this with cypovirus polyhedrin crystals. The resulting structure showed the potential of *in cellulo* crystallization and diffraction.

However, to implement *in cellulo* data collection into a structure pipeline, a method needs to be in place to screen the cells for possible crystals. The screen should require minimum sample preparation and be rapid enough that the cells remain viable. It must be sensitive enough to detect the crystals, even when few in number. It should give direct proof of crystallinity. Available microscopies – bright field, transmission electron and second-harmonic generation – are unsatisfactory in one way or another in meeting all these requirements. Neither are UV fluorescence and micro-electron diffraction completely adequate for detection purposes, owing to the background from high cellular protein concentration in the former and the need for ultra-thin samples in the latter.

With these challenges in mind, Lahey-Rudolph *et al.* overexpressed four test proteins known to crystallize *in cellulo* (Fig. 1 ▸). The crystal-bearing host cells were placed into quartz X-ray capillaries for small-angle X-ray scattering (SAXS), a method designed to work with dilute solutions of macromolecules. The X-ray scattering curves showed distinct peaks (in comparison to the controls), indicating crystalline elements within the scattering volume. The sensitivity was established by recording the scattering curves from a dilution series of the crystal-containing cells. Peak intensities systematically decreased, but the overall trend of the scattering curve remained unaltered. Eight data sets were collected in under 30 min, during which time the cells remained viable.

SAXS can detect weak scattering signals even from noisy backgrounds, like those from living cells. However, it does not allow extraction of unit-cell parameters. To overcome this problem, the authors converted the SAXS data from *I*(*s*) to *I*(2θ) for analysis by XRPD software packages. SAXS data indicate that the Bragg peaks are due to crystallinity, and XRPD analysis allows refinement of unit-cell parameters. Every crystal form has a characteristic diffraction pattern; hence, XRPD is extremely sensitive to crystal polymorphism. By combining the SAXS and XRPD data, Lahey-Rudolph *et al.* harnessed the strengths of both methods for successful identification of crystals *in cellulo*. However, only a few, low-resolution Bragg reflections could be identified, preventing *ab initio* indexing. Initial cell constants and space-group symmetry were, therefore, estimated from reported structures in the Protein Data Bank and then refined from the XRPD data sets (Pawley 1981 ▸).

It would be beneficial to solve the indexing problem. However, even without knowing the cell constants, the method can still be used to study environmental parameters that affect formation of *in cellulo* crystals. This opens the possibility to repurpose overexpression systems for production of crystals rather than proteins. Another positive aspect of *in cellulo* crystallization is that the proteins are in their physiological environment – the living cell. Here they are presumably surrounded by their genuine cofactors and protein partners. Post-translational modifications are also enabled, as seen in the structure solved with *in vivo* crystals of cathepsin B (Redecke *et al.*, 2013 ▸). Furthermore, *in cellulo* data collections are done at room temperature, eliminating structural artifacts induced by cryotemperatures and reducing mosaicity.

The *in cellulo* screening method is a welcome addition to the structural biology toolbox. Ultimately, a better understanding of crystal biogenesis might allow us to transform its occurrence as an incidental or accidental event into an intentional one. *In vivo* crystals may be more common than we realize – under both native and non-native conditions – because we do not screen for them. Without reliable detection methods, they could be hiding in plain sight.

## Figures and Tables

**Figure 1:**
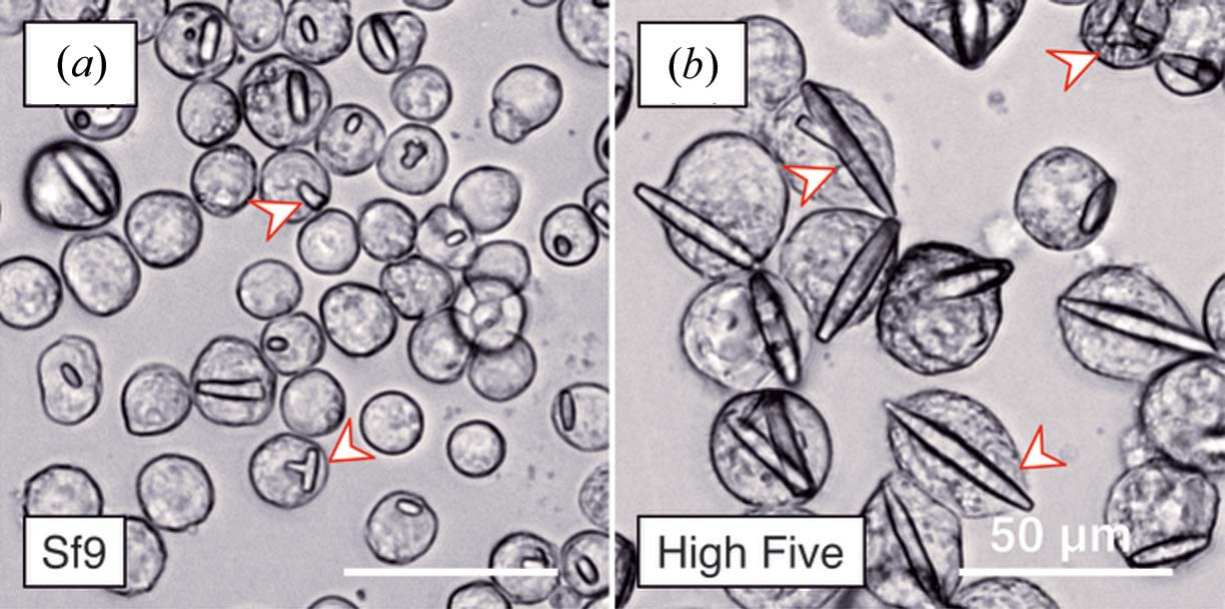
Differential interference contrast light microscopy of intracellular *Neurospora crassa* HEX-1 crystals grown in (*a*) Sf9 and (*b*) High Five insect cells. Red arrowheads highlight selected intracellular crystals. Images courtesy of Lahey-Rudolph *et al.* (2020 ▸).
